# Impaired autophagic and mitochondrial functions are partially restored by ERT in Gaucher and Fabry diseases

**DOI:** 10.1371/journal.pone.0210617

**Published:** 2019-01-11

**Authors:** Margarita M. Ivanova, Erk Changsila, Chidima Iaonou, Ozlem Goker-Alpan

**Affiliations:** Lysosomal and Rare Disorders Research and Treatment Center, Fairfax, VA, United States of America; Univerzitet u Beogradu, SERBIA

## Abstract

The major cellular clearance pathway for organelle and unwanted proteins is the autophagy-lysosome pathway (ALP). Lysosomes not only house proteolytic enzymes, but also traffic organelles, sense nutrients, and repair mitochondria. Mitophagy is initiated by damaged mitochondria, which is ultimately degraded by the ALP to compensate for ATP loss. While both systems are dynamic and respond to continuous cellular stressors, most studies are derived from animal models or cell based systems, which do not provide complete real time data about cellular processes involved in the progression of lysosomal storage diseases in patients. Gaucher and Fabry diseases are rare sphingolipid disorders due to the deficiency of the lysosomal enzymes; glucocerebrosidase and α-galactosidase A with resultant lysosomal dysfunction. Little is known about ALP pathology and mitochondrial function in patients with Gaucher and Fabry diseases, and the effects of enzyme replacement therapy (ERT). Studying blood mononuclear cells (PBMCs) from patients, we provide *in vivo* evidence, that regulation of ALP is defective. In PBMCs derived from Gaucher patients, we report a decreased number of autophagic vacuoles with increased cytoplasmic localization of LC3A/B, accompanied by lysosome accumulation. For both Gaucher and Fabry diseases, the level of the autophagy marker, Beclin1, was elevated and ubiquitin binding protein, SQSTM1/p62, was decreased. mTOR inhibition did not activate autophagy and led to ATP inhibition in PBMCs. Lysosomal abnormalities, independent of the type of the accumulated substrate suppress not only autophagy, but also mitochondrial function and mTOR signaling pathways. ERT partially restored ALP function, LC3-II accumulation and decreased LC3-I/LC3-II ratios. Levels of lysosomal (LAMP1), autophagy (LC3), and mitochondrial markers, (Tfam), normalized after ERT infusion. In conclusion, there is mTOR pathway dysfunction in sphingolipidoses, as observed in both PBMCs derived from patients with Gaucher and Fabry diseases, which leads to impaired autophagy and mitochondrial stress. ERT partially improves ALP function.

## Introduction

Lysosomal storage disorders (LSD) are a group of rare metabolic disorders due to mutations in genes encoding lysosomal enzyme(s) [1]. Gaucher (GD) (OMIM #230800, 231000, 230900) and Fabry (FD) (OMIM #301500) diseases are the two most common LSDs. Glucocerebroside (GC), the last glycolipid in the catabolic pathway of the glycosphingolipid metabolism, accumulates in GD due to deficiency of glucocerebrosidase enzyme (GCase) activity (EC entry 3.2.1.45) [2]. Dysfunctional lysosomes with excessive levels of GC gather in every cell and interfere with cellular pathways outside the lysosomes. Recessively inherited GD presents with a broad spectrum of symptoms encompassing primary nervous system involvement, enlarged spleen and liver, organ dysfunction, anemia, low thrombocyte counts due to bone marrow involvement, and severe skeletal disorder with pain and permanent disabilities [3, 4]. The three clinical subtypes of GD include type 1 (non-neuronopathic or adult), type 2 (acute neuropathic or infantile, where death occurs before age 2 due to rapidly progressive nervous system dysfunction), and type 3 (chronic neuronopathic or juvenile) with chronic neurological problems, pulmonary involvement, organomegaly, and progressive skeletal deformities, such as kyphoscoliosis. The current standard of care, enzyme replacement therapy ERT, is successful for treatment of systemic symptoms. However, ERT becomes less effective during the advanced stages of the disease. In developing countries, ERT is still problematic due to its cost and limitations with enzyme production, distribution, and storage. Another FDA approved therapy for GD is substrate reduction therapy (SRT), and both miglustat and eliglustat act on the ceramide synthesis pathway by decreasing production of the substrate. Neither of the therapies, ERT or SRT, is effective in treating the neuropathic form of GD.

Fabry disease (FD) is an X-linked disorder, where mutations in the *GLA* gene result in a deficiency of the enzyme α-galactosidase A (α–Gal A) (EC entry 3.2.1.22). The α–Gal A hydrolyses the terminal alpha-galactosyl moieties from glycolipids and glycoproteins and its deficiency leads to an accumulation of the substrate, globotriasylceramide (Gb3), in lysosomes. The signs and symptoms of FD are quite heterogeneous, and include renal failure; cardiovascular disease, dermatologic manifestations, ocular complications, auditory, and neurologic complications; all of which are associated with reduced quality of life and early mortality [5]. The life expectancy of male patients with FD, if untreated, is approximately 40–42 years. Similarly, female heterozygotes develop clinical manifestations of varying severity and also have a reduced life span [6]. There are two recombinant α-GalA preparations available for treatment of FD, agalsidase alfa (Replagal, Shire Human Genetic Therapies, Cambridge, MA), which is produced in human fibroblast cell line, and agalsidase beta (Fabrazyme, Genzyme Corporation, Cambridge, MA), which is manufactured using CHO cells; only fabrazyme is approved by the FDA for use in the USA [7].

The major lysosomal pathway, also referred to as autophagy lysosomal pathway (ALP), is a cellular clearance pathway serving as the primary source for organelle turnover and the clearance of unwanted proteins through proteolysis. However, the role of lysosomes is not only limited to housing proteolytic enzymes: they also participate in calcium signaling, trafficking organelles, nutrient sensing, and mitochondria repair [8].

The degradation of damaged mitochondrial proteins through autophagy is called mitophagy. Mitophagy is initiated by the damaged mitochondrion itself, which is ultimately degraded by the macroautophagic pathway to compensate for energy shortage. One of the important stress signals for autophagy activation is environmental stimulation by, for example, pharmacological agents or energy crisis due to intracellular defects of metabolism [9]. The key step in autophagy is the fusion of autophagic vacuoles with lysosomes to form autophagolysosomes, where the macromolecular components are broken down into metabolites that feed into the mitochondria to provide ATP for survival [8, 10]. Therefore, functional lysosomes are essential for autophagy, energy balance, and mitochondrial metabolism. Thus, autophagy and mitochondrial function are the logical targets to study in LSDs, where the primary pathology lies in lysosomal abnormalities.

In this study, we investigated the links between lysosomal abnormalities, ALP function, and the regulation of cellular energy homeostasis in PBMC derived from patients with GD and FD.

## Materials and methods

### Chemicals

Rapamycin (RAP) and 3-Methyladenine (3-MA) were purchased from Sigma-Aldrich (St. Louis, MO, USA). Velaglucerase alfa, a plant-derived recombinant human rhGCase (Shire HGT; Cambridge, MA); and imiglucerase, a rhGCase from overexpressed CHO (Genzyme Cambridge, MA), were obtained from left over vials after reconstitution for patient use.

### Antibodies

LAMP1 was from Abcam (Cambridge, MA, USA); Beclin-1 (2A4), Ser-2448 mTOR, mTOR, LC3A/B, Tfam, GAPDH and β-actin from Cell Signalling (Beverly, MA, USA); and α-tubulin (Ab-2) from Sigma-Aldrich. Glucocerebrosidase (product name; GBA, C1C3) and α-galactosidase A (GLA) antibodies from GeneTex (Irvin, CA, USA).

### Subjects

The diagnosis of GD and FD were confirmed by enzyme assay and molecular analysis. All patients gave a written informed consent for the collection and analysis of their data. Clinical protocol was approved by the ethics committees and data protection agencies at all participating sites (Western Institutional Review Board, WIRB # 20131424). Samples from 29 patients with GD 1 (7 Male: 22 Female, age range 7 to 77 years, mean 50±17), 5 patients with GD3 (2 Male: 3 Female, age range; 12 to 38 (mean 16±11), and 21 patients with FD (16 Male: 5 Female, age range 9 to 61, mean 31±16) were studied. Patients with GD were treated with velaglucerase alfa (Shire, Biotechology Company, Dublin, Ireland). FD patients were treated with agalsidase beta (Genzyme Therapeutics, Cambridge, MA). The demographics, genotypes and other relevant clinical information are summarized in S1 Table. Samples were collected prior ERT infusion, except experiments comparing pre- and post- infusion.

### PBMC isolation and cell culture experiments

Peripheral blood mononuclear cells (PBMCs) were purified from patient blood samples using Lymphoprep (Stemcell Technologies) and SepMate tubes (Stemcell Technologies, Vancouver, Canada). Lymphoprep was added to the lower compartment of the SepMate tube. Blood was mixed with PBS + 2% FBS in a 1 to 1 ratio, then layered on top of Lymphoprep following the company protocol. Samples were centrifuged at 800g for 20 min at 18°C with the brake off. The upper plasma layer was discarded. The PBMCs layer was removed carefully then washed with PBS and centrifuged at 300g for 8’ at room temperature between each wash. The pellet was stored at -20°C or isolated PBMCs used immediately following the experiments. 10X10^6^ fresh lymphocytes sample, ID W313715036917, was received from HemaCare Corporation (Van Nuys, CA, USA). THP-1(TIB-202) cells were purchased from ATCC (Manassas, VA, USA) and maintained following the manufacture protocol. PBMCs and THP-1 cells were treated with human recombinant proteins rhGCase or rhα-Gal A, rapamycin (RAP), or a combination of RAP and 3MA in phenol red-free RPMI media with 10% FBS.

### Protein isolation and western blot analysis

Whole-cell extracts (WCE) were prepared in radioimmunoprecepitation (RIPA) buffer. Protein concentrations were determined using the BCA Protein Assay Kit (ThermoFisher Scientific, Rockford, IL, USA). 30–40 μg of WCE were separated on mini protein TGX stain free gel (Bio-Rad, Hercules, CA, USA) and electroblotted using the Trans-Blot Turbo Midi PVDF Transfer Packs (Bio-Rad). The ChemiDoc MP Imaging system (Bio-Rad) was used to visualize and quantitate optical density (IOD) for each band. The IODs of bands of interest were normalized to the loading control and used in the same blot: e.g. Ponceau staining bands, α-tubulin or b actin and the normalized value of the controls was set to 1 for comparison between separate experiments.

### Autophagy assay

The Cyto-ID Autophagy detection kit (ENZ-51031-K200, Enzo Life Sciences, Farmingdale, NY, USA) was used according to the manufacture’s protocol to quantify autophagic vesicle number by cationic amphiphilic tracer (CAT) fluorescence dye and Hoechst 33342 dye as an index of nucleus. PBMCs were stained for 30 min with CAT and Hoechst then washed with PBS. Fluorescence was measured in triplicates using a SpectraMax M2 microplate reader (Molecular Devices, Sunnyvale, CA, USA). Data was normalized to a control (healthy subjects) or untreated cells.

### Measurement of lysosome levels

The LysoTracker Red (LifeTechnology, ThermoFisher, Rockford, IL, USA) assay was used as briefly described. LysoTracker (50 nM) was added to the cultured PBMCs as a fluorescent acidophilic probe for the labelling of the acidic organelles. After 30 min staining, cells were stained with Hoechst, washed 3 times with PBS, then re suspended in 96-well black plates with clear bottom. The red fluorescence of LysoTracker was measured in triplicates using a SpectraMax M2 microplate reader with excitation wavelength: 577 nm; emission wavelength: 590 nm (Molecular Devices, Sunnyvale, CA, USA). Data was normalized to control (healthy subjects) or untreated cells.

### Mitochondrial characterization

The MitoTracker Red CMXRos mitochondrial kit (ThermoFisher scientific) was used according to the manufacture protocol to quantify mitochondrial volume. Nuclear Hoechst dye was used as index for cells contents. Cells were stained with a fluorescence probe for 30 min and after washed with PBS. The MitoTracker Red CMXRos signal was measured in triplicates using the SpectraMax M2 microplate reader with excitation/emission 577–590.

### Mitochondrial membrane potential assay

The mitochondrial membrane potential was determined using the Mito-ID membrane Potential Cytotoxicity Kit (Enzo Life Sciences, Farmingdale, NY, USA). In energized inner membrane, the mitochondria produced an orange fluorescence signal. If cells exhibit a shift from orange to green fluorescence: mitochondrial function becomes increasingly compromised. Freshly isolated PBMCs were stained with mito-ID membrane potential dye loading solution in black, clear-bottom 96-well tissue culture dishes for 30 min. After incubation, the resulting fluorescence was visualized by fluorescent microscopy (Evos Digital microscope, Evos, Hatifiled, PA, USA).

### ATP assay

For the measurement of ATP levels, the CellTiter-Glo luminescent cell viability assay (Promega, Madison, WI, USA) was used. To measure relative levels, the cells were plated in 96-well white plates following the manufacturer protocol. After treatments and the autophagy assays, the same volume of CellTiter-Glo Reagent was added directly to the samples and incubated for 15 min. To measure levels of ATP after freezing cell pellets, cell pellets were boiled for 5 min in 100 μl of nuclease-free water, and were centrifuged. Then, the cell lysates were chilled in iceblocks for subsequent analysis [11]. Protein concentrations were determined using BCA protein assay. Following manufacture’s protocol, ATP standard and the cell lysates (10 ng) were analyzed by measuring bioluminescence signal in a Gemini microplate reader. Samples were run in triplicates with a total of 4–11 separate experiments (biological replicates) and performed for statistical evaluation (Student’s two-tailed t-test).

### RNA isolation and quantitative real-time-PCR (qPCR)

RNA was extracted from patient and control PBMCs using the RNeasy Mini Kit from Qiagen (Valencia, CA, USA). The SuperScript VILO cDNA synthesis kit (Invitrogen, ThermoFisher, Rockford, IL, USA) was used to reverse-transcribe RNA using random hexamers. Samples were prepared using PowerUp SYBR Master Mix (Invitrogen, ThermoFisher, Rockford, IL, USA). Transcript expression analysis was determined between control PBMCs and PBMCs derived from Gaucher or Fabry patients. Individual samples were run in triplicate. Human mRNA transcript levels of LAMP1, LC3A/B, SQSTM1/p62, DDIT3 and HSPA5 as compared to loading controls GADPH and HPRT were measured using StepOnePlus Real-time PCR System (Life Technologies, (Invitrogen, ThermoFisher, Rockford, IL, USA)). The primers were purchased from LifeTechnology (S2 Table). Sequence of NRF1, Tfam and CytC primers have been previously published [12]. Sequences of SQSTM1/p62 primers have been previously published [13]. Analyses and fold differences were determined using the comparative CT method. Fold change was calculated from the ΔΔCT values with the formula 2^−ΔΔCT^ relative to mRNA expression in control donors.

### Mitochondrial/Nuclear DNA ratios

The relative mitochondrial content of PBMCs derived from controls’ and patients’ blood was determined using PowerUp SYBR qPCR by measuring the ratio of mitochondrial- encoded nicotinamide adenine dinucleotide dehydrogenase-1 or 2 (mt-ND1)/ nuclear-encoded GAPDH [12, 14]. For nuclear and mitochondrial DNA quantification, 10 ng and 0.1 ng DNA were used as templates, respectively. Each sample was analyzed in triplicates. Relative mitochondrial copy number to nuclear copy number (mtDNA copy number) was assessed by a comparative Ct method, using the following equation: ΔCt mitochondria/nuclear = Ct mitochondria–Ct nuclear. The fold-change relative to control samples was calculated using the following equation: 2^(−ΔΔCt)^ mitochondria/nuclear, where ΔΔCt mitochondria/nuclear = ΔCt control mitochondria/nuclear –ΔCt mitochondria/nuclear of each sample from different treatment and time groups [14]. Values represent mean fold change ± SEM.

### Enzyme-linked immunosorbent assay (ELISA)

SQSTM1/p62 expression level was measured with a PathScan Total SQSTM1/p62 Sandwich ELISA kit, PathScan Total mTOR Sandwich ELISA kit, PathScan Phospho-mTOR (Ser2448) mTOR Sandwich ELISA kit, (Cell Signaling Technology, #7814C, #7974C, #7976 Danvers, MA, USA) according to the manufacturer’s instructions. The PBMCs were purified and frozen immediately after blood collection. The cells were lysed with lysis buffer from the kit and 50 μg (SQSTM1/p62 ELISA) and 75 μg (Ser2448 and mTOR) of protein lysates were used for assay. All samples were assayed in duplicate and data was represented from three separate experiments. Values represent the average of percentage change to healthy control ± SEM.

### Statistical analysis

Statistical analysis was performed using Student’s T test with a 1-tailed distribution and 2 samples of equal variance comparing control & one group, and 1-way ANOVA followed by Brtlett’s, Kruskal-Wallis or Dunn’s multiple comparison tests using GraphPad Prizm (GraphPad, San Diego, CA, USA).

## Results

### Deficiency of β-glucocerebrosidase activity impairs formation of autophagic vesicles in PBMCs

**Fig 1:**
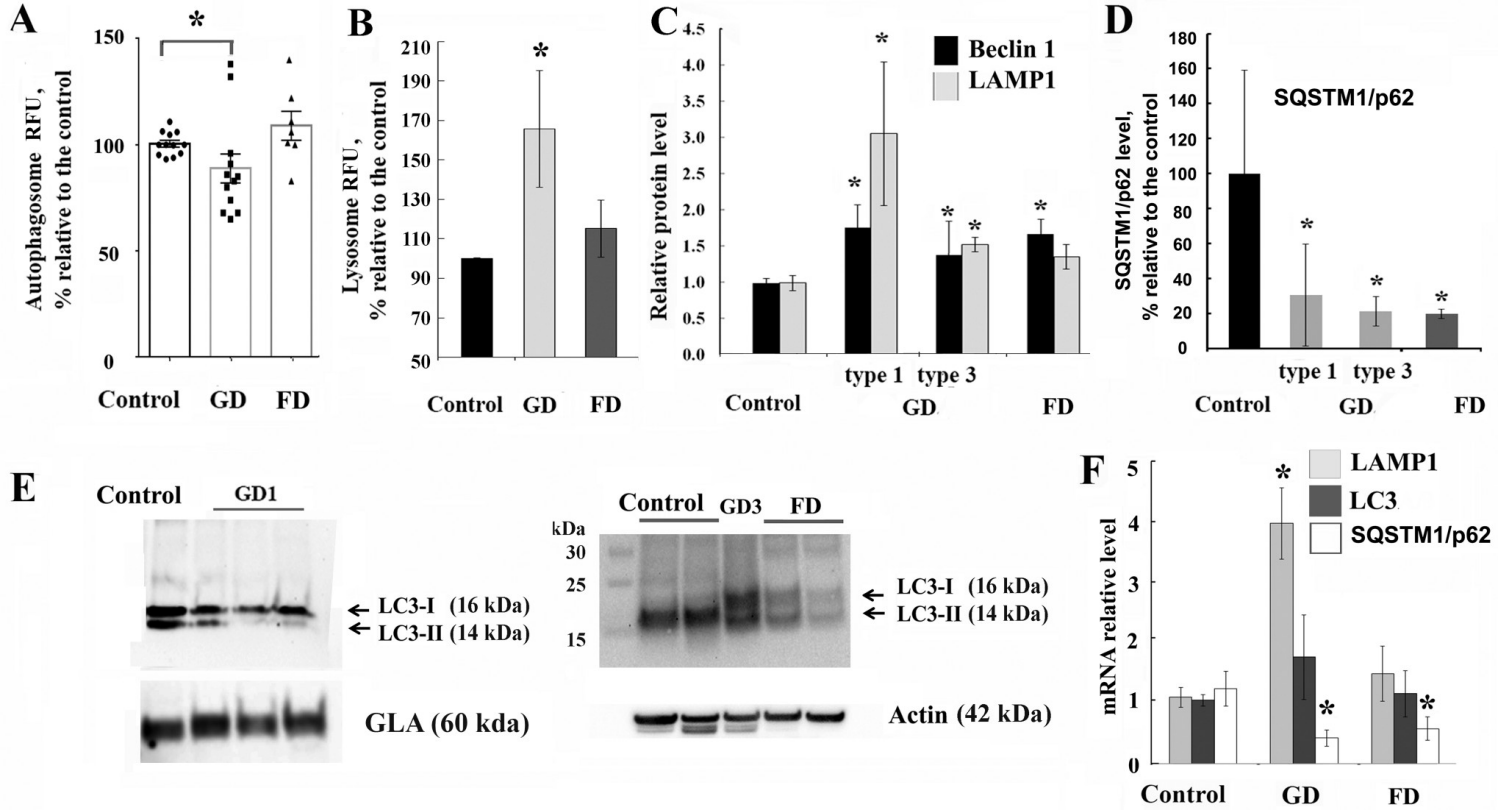
Autophagy-lysosomal complex is affected in GD and FD. **(A)** PBMCs from healthy subjects (control), patients with GD (types 1 and 3) and FD were stained with Cyto-ID autophagy detection kit. The graph shows the relative levels of autophagosome vesicle number in control, GD and FD groups. Values were normalized to the control group. Each dot represents a patient sample.*p<0.*05* One-way ANOVA, p = 0.016 ANOVA with Bartlett’s multiple comparisons test. **(B)** GD, FD and control PBMCs were stained with LysoTrackerRed to measure lysosomal accumulation. Values were normalized to the control group. GD (n = 5), FD (n = 6) and control (n = 10). *p<0.*05*. **(C)** Quantification of Beclin 1 and LAMP1 protein level from control (n = 10), GD1 (n = 5), GD3 (n = 4) and FD (n = 8) samples after western blot. The protein level was measured relative to total protein level. Values were normalized to the control group. *p<0.*05* Student’s T-Test. **(D)** SQSTM1/p62 level in GD type 1 and type 3 (n = 11), FD (n = 5) and control (n = 5) samples were measured with a SQSTM1/p62 ELISA kit. SQSTM1/p62 level was measured in triplicates, on three biological repeats. Values normalized to control group. *p<0.*05* Student’s T-Test. **(E)** Representative western blot showing LC3-I and LC3-II levels in PBMCs from GD (Type 1 and 3), FD and control samples. (**F**) Q-PCR was performed to determine relative LAMP1, LC3A/B and SQSTM1/p62 mRNA expression levels in GD and FD PBMCs. RNA level was measured in triplicates, n = 5 or 10 samples analysed each group. All data were expressed as S.E.M. and * p<0.*05 vs control*.

To examine the implications of GCase or α-GalA deficiency on ALP function, the level of autophagosomes was studied *in vivo* using the Cyto-ID Autophagy kit which measures autophagy with a cationic amphiphilic tracer dye labelling the vacuoles associated with the autophagy pathway. PBMCs were isolated from healthy subjects or patients with Gaucher and Fabry diseases, and then were stained immediately after isolation. The percentage of autophagic vacuoles was decreased in GD compared to healthy subjects (Fig 1A), suggesting that accumulation of GC in lysosomes inhibits the number of autophagosomes. The presence of L444P/L444P, N370S/R463C, N370S/L444P, N370S/N370S, or N370S/R296Q *GBA* mutations is associated with reduced basal autophagosome levels by 10–40%, except for one patient with N370S/N370S and one with N370S/L444P mutation (S1 Fig). As we expected, a decrease in the GCase enzyme activity resulted in an increased lysosome accumulation in PBMCs (Fig 1B). This data is consistent with the well-known lysosomal pathology of GD [15, 16]. Contrary to GD, in PBMCs from patients with FD, there was no inhibition of autophagosome vesicle level (Fig 1A), except in one sample from a male patient with W95L mutation (S1 Fig) and no accumulation of lysosomes (Fig 1B).

### PBMCs derived from GD or FD patients have elevated levels of Beclin1, LAMP1 and supressed level of LC3-II and SQSTM1/p62

In the autophagy-lysosomal pathway, the lysosome marker LAMP1, autophagy markers Beclin 1, SQSTM1/p62 and LC3-I/II proteins are critical for maintaining the autophagy function in cells [17]. Western blot analysis showed an increase in Beclin1 levels in PBMCs from patients with GD and FD, whereas LAMP1 level only increased in GD samples compared to healthy controls (Fig 1C and S1 Fig). Similar to GD1, in GD3 samples with a L444P/L444P mutation, we also observed an increase in Beclin 1 and LAMP1 (Fig 1C and S1 Fig). Sequestosome 1 (SQSTM1/p62) binds autophagosomal membrane protein LC3, bringing p62-containing protein “cargo” to the autophagosome [18]. ELISA assay showed a reduced level of SQSTM1/p62 in PBMCs derived from patients with GD or FD (Fig 1D). LC3-I, a cytoplasmic isoform of LC3, can be converted to LC3-II which then binds to the autophagosome membrane and is considered a marker of autophagic activity [19]. Western blot analysis showed cytoplasmic accumulation of LC3-I and significantly decreased levels of LC3-II in GD and FD PBMCs (Fig 1E and S1 Fig), although the total expression of *LC3*, as measured by mRNA levels, was indistinguishable among control, GD or FD PBMC (Fig 1F), suggesting the inactivation of constitutive autophagy. In addition to increasing LAMP1 protein levels, the expression of *LAMP1* mRNA was higher in GD than in PBMCs from healthy donors or FD patients (Fig 1F). LC3 mRNA levels showed no difference in PBMCs derived from patients with GD or FD compared to healthy controls (Fig 1F). The expression level of *SQSTM1/p62* mRNA was lower in GD1 and FD than in PBMCs from healthy donors, this result commensurates with ELISA assay for SQSTM1/p62 protein (Fig 1F).

### Lysosomal dysfunction hampers rapamycin induced autophagy activation

**Fig 2:**
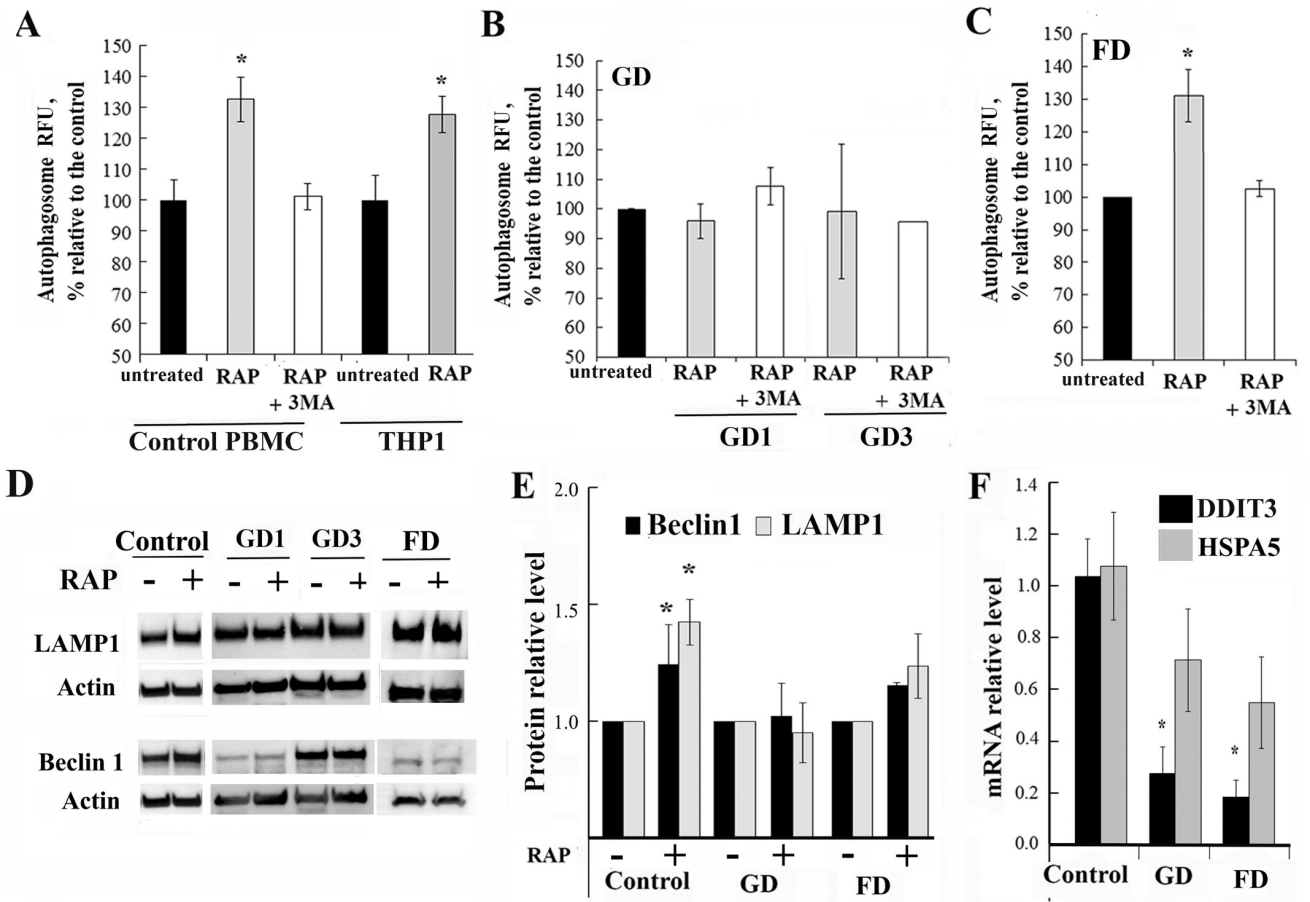
The effects of mTOR inhibitor rapamycin on autophagy in GD and FD. **(A-C)** PBMCs from healthy controls (A), GD (B), and FD (C) were treated with the 10 nM rapamycin (RAP) alone or in combination with 20 μM 3MA for 3h. Then, PBMCs were stained with Cyto-ID autophagy detection kit. Samples were measured in triplicates, control group n = 5, GD1 n = 11, GD3 n = 3, FD n = 5. All data were expressed as S.E.M. and * p<0.*05 vs control*. **(D)** Representative western blot showing Beclin1 and LAMP1 protein expression in GD (N370S/L444P and L444P/L444P mutations), FD, and control samples after 3h treatment with 10 nM rapamycin. **(E)** Quantification of relative levels of Beclin1 and LAMP1 normalized to actin from separate experiments in which the control were untreated PBMCs from the same group. n = 5–7 samples analysed each patient group. Membranes were re-probed with actin or total protein for normalization.* p<0.*05 vs untreated samples*. **(F)** Q-PCR was performed to determine relative DDIT3 and HSPA5 mRNA expression levels in GD and FD samples. The RNA level was measured in triplicates, n = 5 or 6 samples analysed each group. All data are expressed as S.E.M. and * p<0.*05 vs untreated samples*.

To assess the involvement of mTOR pathway in the pathophysiology of Gaucher and Fabry diseases, PBMCs from patients and healthy controls were treated with rapamycin for 3h because short incubation with rapamycin specifically abolishes mTORC1, but not mTORC2 activity [20–22]. It is well known that rapamycin induces autophagy through inhibition of mTOR pathway by disrupting mTORC1-protein complex formation and inhibiting phosphor-mTOR (S2448) [23]. Inhibition of mTOR by rapamycin was confirmed by western blot analysis with phospho-mTOR (S2448) antibodies (S2 Fig). Rapamycin reduced phosphorylation of mTOR S2448 in PBMCs derived from healthy controls and primary fibroblasts after 3 h treatment (S2 Fig). The rapamycin effect could be blocked by 3-methyladenine (3-MA), which inhibits autophagy by inhibiting class III P13K. An increase in autophagic vesicle level was observed in control PBMCs, human monocytic cells (THP1) and FD PBMCs after treatment with rapamycin and was abolished by 3-MA (Fig 2A and 2C). However, there was no difference in autophagy between untreated and rapamycin-treated GD samples (Fig 2B). Basal Beclin-1 level was elevated in untreated GD and FD PBMCs (Fig 1C). However, it did not increase further from already elevated basal level after the 3h incubation with rapamycin in contrast to a ~1.3-fold increase observed in healthy controls (Fig 2D and 2E and S2 Fig). Basal levels of the LAMP1 were elevated in GD, but not in FD PBMCs (Fig 1E and 1F). Similar to Beclin-1, 3h treatment with 100 nM rapamycin did not cause an increase in LAMP1 in GD or FD PBMCs in contrast to control cells (Fig 2D and 2E and S2 Fig), suggesting that the mTOR pathway was dysfunctional.

Next, we measured a phosphorylation of mTOR at position S2448 and mTOR level in untreated PBMCs. Phosphorylation at position S2448 is associated with the activation of mTOR and of the PI-3 kinase pathway [24]. S2448 is phosphorylated by S6K, which directly reflects amino acid and nutrient status. In response to cellular stress, phosphorylation of mTOR at S2448 is reduced and the brake imposed on autophagy is released. S2448 mTOR level was significant decreased in untreated GD PBMCs (S2 Fig). This data supports that lysosomal dysfunction downregulates autophagic processes and the mTOR pathway in GD and FD PBMCs.

### DDIT3 transcription is supressed in Gaucher and Fabry diseases

mTOR pathway not only regulates autophagy, but also controls the transcription of DNA damage-inducible transcript 3, DDIT3, (also known as (CHOP, C/EBP homologous protein) [25, 26]. DDIT3 in turn regulates the expression of Heat Shock 70 kDa Protein 5 (Glucose-Regulated Protein) HSPA5. DDIT3 and HSPA5 play a role in stress-induced autophagy [27]. Therefore, we examined the expression of DDIT3 and its target gene, HSPA5. Both, in GD and FD PBMCs, DDIT3 mRNA levels were downregulated regardless of the type of *GBA* or *GLA* mutations (Fig 2F), whereas the levels of HSPA5 showed a downward trend in patient samples, but did not reach statistical significance. This data was consistent with the observation that the mTOR pathway is affected in GD and FD PBMCs.

### ATP synthesis is impaired in GD and FD

**Fig 3:**
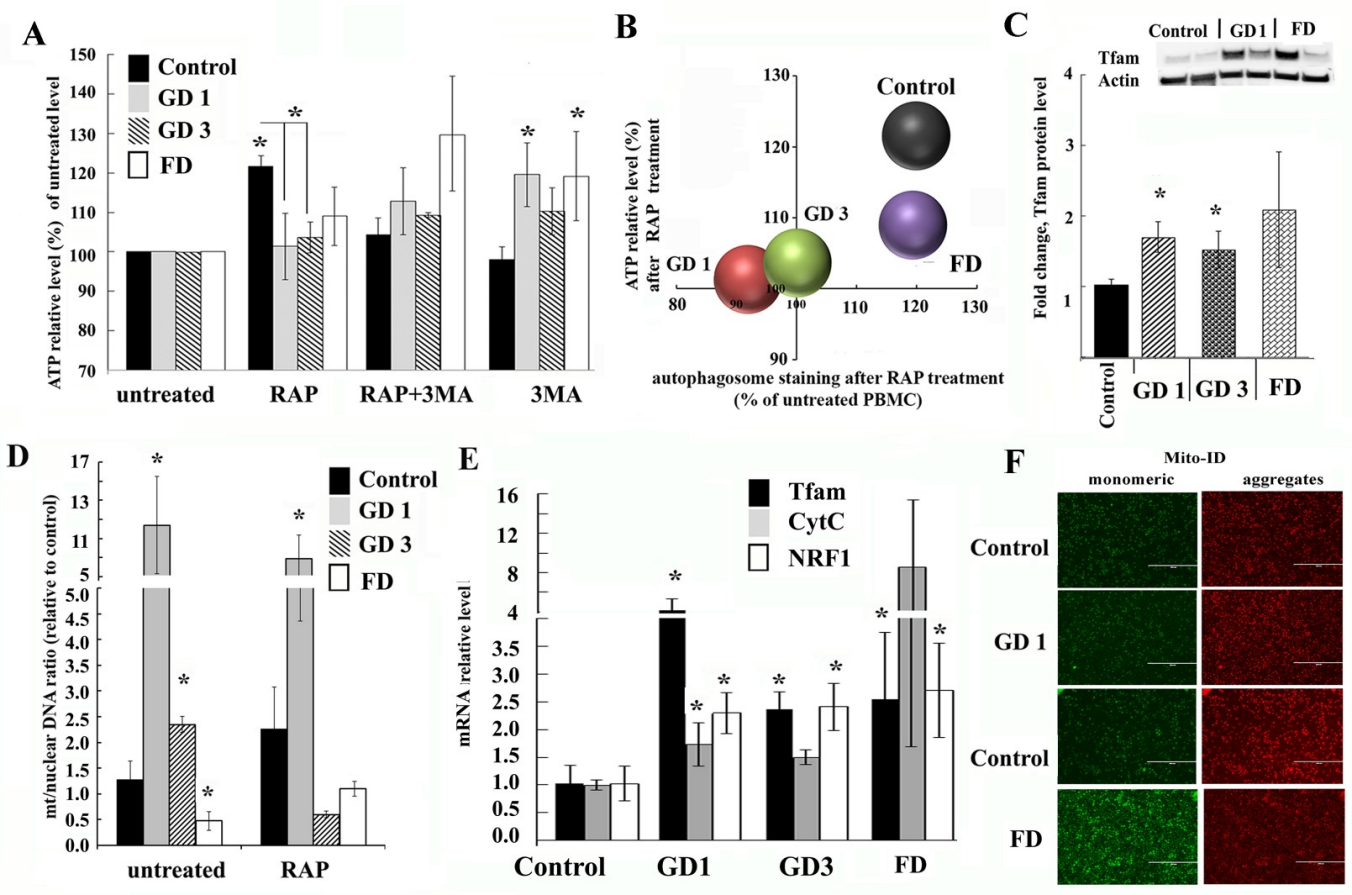
Mitochondrial function is affected in GD and FD. **(A)** Effect of rapamycin (RAP) treatment on ATP levels. PBMCs from healthy subjects, GD and FD were treated for 3h with the 10 nM RAP alone, 20 μM 3MA or a combination. ATP levels were measured using CellTiter-Glo assay. Samples were measured in triplicates, control group n = 4, GD1 n = 6, GD3 n = 3, FD n = 4. All data were expressed as S.E.M. and * p<0.*05 vs untreated control*. **(B)** A scatter diagram comparing autophagosome volume to ATP levels in response to 10 nM RAP treatment for 3h. **(C)** Quantification of Tfam protein level from control (n = 10), GD1 (n = 10), GD3 (n = 3) and FD (n = 6) samples after detection by western blotting. Quantification of relative level of Tfam normalized to actin. Insert: representative western blot showing Tfam protein expression in healthy subjects (control), GD1 and FD samples. *p<0.*05 control vs GD* (Student’s T test, One-way ANOVA with Kruskal-Wallis test) and *p = 0*.*037 controls vs*. *GD* (Dunn’s multiple comparisons test). **(D)** Analysis of mitochondrial biogenesis. PBMCs from healthy subjects (Control), GD1, GD3 and FD were treated with the 10 nM rapamycin for 3h. The data is the ratio of the mitochondrial encoded gene ND1 to nuclear-encoded GAPDH as determined by qPCR. Untreated PBMCs from healthy subjects served as the control and an average of their values was set as 1. Data were expressed as S.E.M. and * p<0.*05* **(E)** Q-PCR was performed to determine relative Tfam, CytC and NRF1 mRNA expression levels in GD1 and FD samples. RNA level was measured in triplicates, n = 3–4 samples analysed each group. All data were expressed as S.E.M. and * p<0.*05 vs control group*. **(F).** Fluorescence images of ΔѰm. The mitochondria of freshly isolated PBMCs from patients with GD, FD and healthy controls were stained with Mito-ID Membrane Potential reagent, and visualized by fluorescence microscopy. Green fluorescence color stain mitochondria with a low membrane potential, highly polarized mitochondria exhibit red color.

Primary autophagosome-lysosomal defects could lead to the accumulation of dysfunctional mitochondria and compromise energy metabolism [28, 29]. To examine the mitochondrial function in patients, we first measured the ATP levels in patients derived PBMCs. While there was some variability in relative ATP levels in GD1 and FD PBMCs, overall levels were similar to that of control PBMCs (S3 Fig). To examine the effect of the mTOR pathway on the ATP level, PBMCs were treated with mTOR inhibitor, rapamycin, because rapamycin not only activate autophagy through inhibition of mTOR [20], but also control mitochondrial function [30]. The ATP level was increased in normal PBMCs after treatment with rapamycin (Fig 3A); however, it remained unchanged in GD and FD cells. On the other hand, ATP levels were paradoxically increased in GD and FD PBMCs treated with autophagy inhibitor 3-MA (Fig 3A). Comparative scatterplot analyses revealed a positive correlation between autophagosome number and ATP levels in rapamycin-treated PBMCs (Fig 3B). This data indicates that lysosomal dysfunction suppresses not only autophagy dynamics, but also disrupts mTOR regulation of ATP synthesis in PBMCs from GD and FD patients.

### Lysosomal dysfunction impairs mitochondrial function in PBMC derived from GD and FD patients

We further investigated whether mitochondrial activity and mitophagy were affected *in viv*o. Mitochondrial transcription factor A (Tfam) is crucial for the initiation of mitochondrial transcription and DNA replication [31]. Mitochondrial DNA (mtDNA) does not freely float in mitochondrial matrix, it is covered by Tfam protein to maintain the proper shape and control the copy number [32]. Tfam levels were increased in PBMCs from patients with GD and FD, while there was variability among individual samples (Fig 3C and S3 Fig).

Since lysosomal dysfunction in general inhibits mitophagy [33], we examined the mt/nuclear DNA ratio as an index of mitochondrial biogenesis. The mt/nuclear DNA ratio was determined by qPCR using primers for mt-ND1 or mt-ND2 as mitochondrial genes and GAPDH as a nuclear gene. The mt/nuclear DNA ratio was increased in both GD1 and GD3, but decreased in FD (Fig 3D). Rapamycin treatment did not affect the mtDNA copy number in either of the healthy controls, GD1, or FD cells (Fig 3D), whereas it reduced mtDNA copy number in GD3, suggesting activation of mitophagy.

Because Tfam protein level was elevated in GD and FD cells, the levels of Tfam mRNA were also studied. Both, in GD and FD PBMCs, the Tfam mRNA expression level was increased (Fig 3E). To determine the mechanism of Tfam activation, the transcription factor controlling Tfam expression, nuclear respiratory factor 1 (NRF1) was examined [34]. Also, we included the nuclear-encoded mitochondrial gene cytochrome c (CytC) because NRF1 also controls expression of CytC mRNA. NRF1 mRNA level was increased in PBMCs derived from Gaucher and Fabry patients (Fig 3E). The CytC level was elevated in FD and to a lesser extent in GD1 PBMC. Our findings indicate that the transcription of Tfam and mitochondrial biogenesis were both activated in GD and FD PBMCs.

Mitochondrial function relies on the mitochondrial membrane potential (ΔѰm). Therefore, we measured ΔѰm using Mito-ID dye in healthy controls, GD1 and FD PBMCs. Healthy mitochondria that have high ΔѰm uptake dye inside the mitochondria and emit red fluorescence at 590 nm, damaged mitochondria with low ΔѰm emit green fluorescence. FD PBMCs exhibited stronger green fluorescence than control cells, indicating increasing level of depolarized mitochondria’s (Fig 3F). GD PBMCs exhibited similar patterns to healthy control levels of ΔѰm. We conclude that a primary defect of autophagy-lysosomal system has a secondary impact on the mitochondrial function.

### Effect of ERT on autophagy and mitochondrial functions

**Fig 4:**
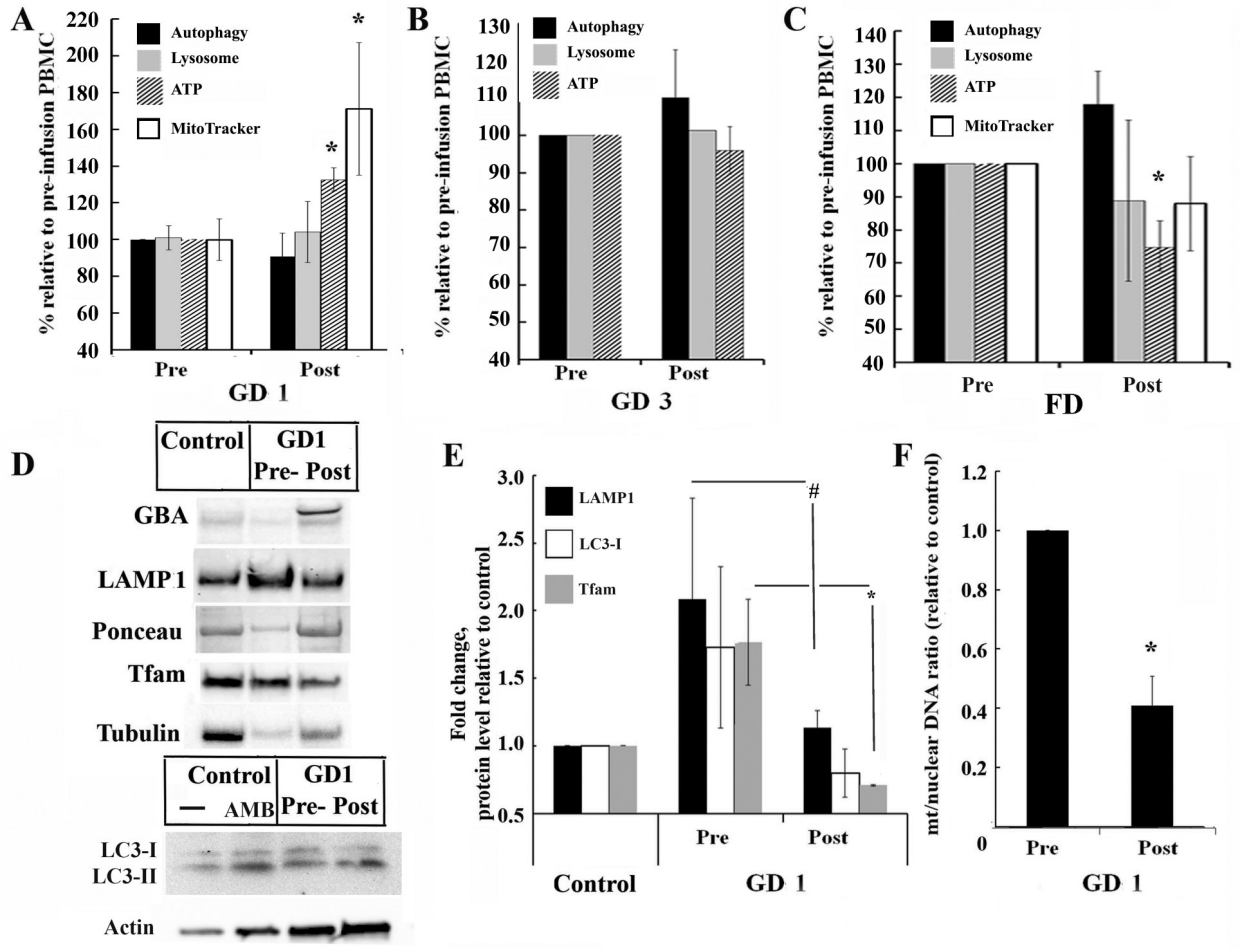
Assessment of autophagy and mitochondrial functions after ERT. **(A, B and C)** Autophagy vacuole number, lysosome, cellular ATP and mitochondrial activity (MitoTracker) levels were measured immediately following ERT treatment. Blood was collected before (Pre) and after (Post) ERT infusion and PBMCs were isolated from GD1 (A), GD3 (B) and FD (C) patients. Values represent post-infusion to pre-infusion levels. Samples were measured in triplicates, n = 3. All data were expressed as S.E.M. and * p<0.*05 vs pre-infusion*. **(D)** Representative western blot (30 μg WCE) shows GBA, LAMP1, LC3-I/II and Tfam protein expression in PBMCs of healthy and GD1 subjects before and after ERT infusion. LC3 western blot representing protein expression in untreated (-) or treated with 100 μM ambroxol (AMB) PBMC derived from healthy subjects (control) and GD1 samples before and after ERT infusion. **(E)** Quantification of LAMP1, total level of LC3I and Tfam normalized to protein bands on Ponceau staining from n = 3–4 separate experiments in which control were PBMCs from healthy control group. * p<0.*05 vs pre-infusion samples* (Student’s T test). *# p = 0*.*013 vs pre-infusion samples (*F test to compare variance). **(F)** Relative amount of mitochondria in GD1 patient pre- and post-infusion. Shown is the ratio of the mitochondrial encoded gene ND1 to nuclear-encoded GAPDH as determined by qPCR. Samples were measured in triplicates, n = 4. All data were expressed as S.E.M. and * p<0.*05 vs pre-infusion samples* (Student’s T test).

We assessed whether ERT could reverse lysosomal dysfunction and normalize mitochondrial function in both *in vivo and in vitro* models. Immediately before and after ERT infusion, blood was collected from patients and autophagic vacuole components, lysosome volume, mitochondrial activity and ATP were measured. The rate of autophagosome formation and lysosomes in GD1 and GD3 PBMCs did not change after rhGCase treatment (Fig 4A and 4B) or in FD PBMCs after rhα-Gal A infusion (Fig 4C). The level of ATP and mitochondrial activity were increased after ERT administration in patients with GD1 (Fig 4A). No change was observed in GD3 and an opposite trend was observed in patients with FD, where ATP levels slightly decreased after the infusion (Fig 4C). Moreover, to further examine if rhGCase infusion may render activation of autophagy and mitophagy in GD, we measured protein level of LAMP1, LC3A/B I-II, Tfam and mtDNA copy number. Western blot analysis demonstrated robust uptake of recombinant GCase by PBMCs after infusion (Fig 4D and S4 Fig). The level of LAMP1 was decreased in PBMCs after enzyme treatment to a level similar to healthy controls, as shown in Fig 4D and 4E and S4 Fig. Control PBMCs treated with ambroxol (AMB) have been used as a positive control converting LC3-I form to the lower migration form LC3-II (Fig 4D). The decreased level of LC3-I was observed in cells after ERT (Fig 4D and S4 Fig). ERT infusion significantly decreased both Tfam level and mtDNA copy number and brought these parameters closer to the level of healthy controls (Fig 4E and 4F, S4 Fig).

### Treatment with recombinant human enzymes improve autophagy

**Fig 5:**
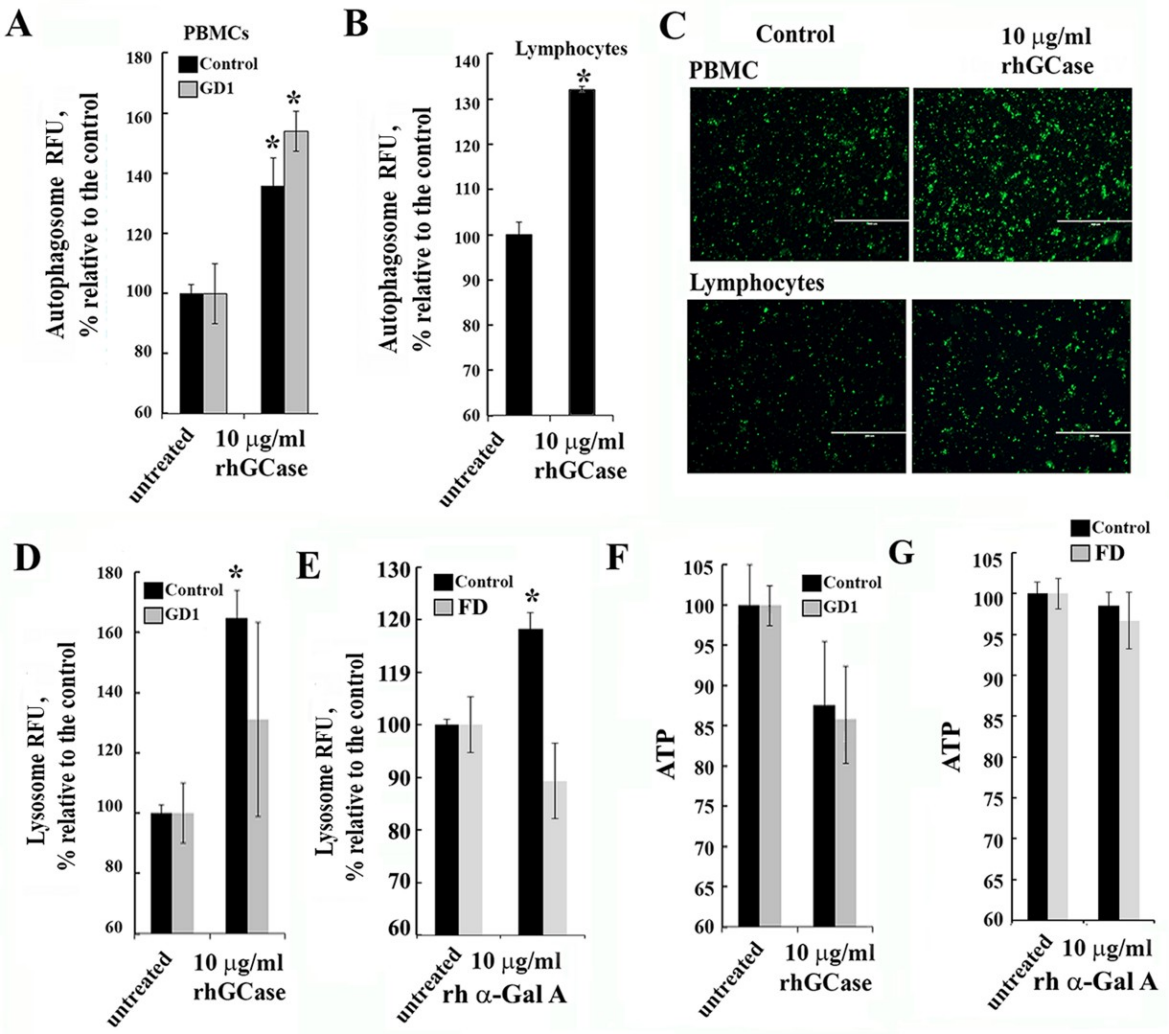
Effect of recombinant enzyme on the ALP pathway and ATP levels. **(A-B)** Autophagy in PBMC (A) or lymphocytes (B) following recombinant GCase treatment. PBMCs or lymphocytes from healthy subjects (control, black bar) and GD1 (grey bar) patients were treated for 3h with 10 μg/ml rhGCase and stained with Cyto-ID autophagy detection kit. **(C)** Autophagosome staining of PBMCs and lymphocytes (from healthy subjects) incubated with or without 10 μg/ml rhGCase. Staining was performed using Cyto-ID autophagy kit (green color). **(D and E)** Effect of recombinant GCase and α-Gal A on lysosomes. PBMCs from healthy (black bar) and GD1 (grey bar) individuals were incubated for 3h with the 10 μg/ml rhGCase or α-Gal A and stained with lysosomal dye, LysoTracker Red. **(F)** ATP level in healthy (control) and GD type 1 PBMCs before and after 3h rhGCase treatment. **(G)** ATP level in control and FD PBMCs before and after 3h α-Gal A treatment. All samples were measured in triplicates, n = 3–4 patients from each group. All data were expressed as S.E.M. and * p<0.*05 vs untreated samples*.

Considering the lack of clarity concerning the effects of ERT on autophagy and mitochondrial function in patients with GD, we treated PBMC with rhGCase *in vitro*. To determine optimal concentration of rhGCase for treatment, THP1 cells were treated for 3h with two commercial rh GCase enzymes: velaglucerase alfa and cerezyme. Based on the enzyme activity measurements and autophagosome vesicle number, the rhGCase concentration of 10 μg/ml was chosen (S5 Fig). Treatment with rhGCase revealed tendency to increase autophagosomal vesicle number in healthy and GD PBMCs and lymphocytes derived from healthy group (Fig 5A, 5B and 5C), suggesting that the increasing levels of GCase enzyme activity is responsible for autophagy activation. Treatment with rhGCase or α-Gal A increased lysosomal activation in control PBMCs (Fig 5D and 5E). However, rhGCase did not increase lysosomal activity in GD samples, which could be because lysosomal levels had already been saturated in GD (Fig 5D). α-Gal A did not increased lysosomal activity in FD PBMCs (Fig 5E). ATP levels were reduced in GD1 samples by 10% compared with untreated control, but after ERT we noticed increasing ATP levels in GD1 PBMCs (Fig 5F). Given that the reduction of ATP levels occurs during ERT infusion in patients with FD, we treated Fabry PBMCs with 10 μg/ml of recombinant α-Gal A *in vitro*. A decrease in ATP levels was not observed during treatment of PBMC *in vitro* (Fig 5G). These results suggest that the effects of ERT on autophagy or mitochondrial process in patient blood cells are complicated and do not directly follow uptake of recombinant enzyme *in vitro*.

## Discussion

Lysosomes provide a control system for the clearance of cellular material and products of the energy metabolism [28]. When sphingolipids accumulate in the lysosomes, the pH increases and mediates lysosomal destabilization. However, the molecular mechanisms leading to malfunction of ALP and energy pathways in LSDs still not fully understood [17, 35, 36]. Studies examining autophagy-lysosomal and mitochondrial functions in LSDs mostly relied on *in vitro* studies and animal models, often focusing on mechanisms of neurodegenerative pathology [36–38]. Here we present the *in vivo* study assessing ALP and mitochondrial regulation in two distinct lysosomal storage disorders: Gaucher and Fabry diseases. We report that lysosomal abnormalities in PBMCs from patients with GD or FD lead to dysfunctional autophagy and disturb the energy balance and mitochondrial function, despite the fact that the type of substrate accumulating in lysosomes differs greatly between the two disorders.

The dynamics of autophagy includes the autophagosome formation, the fusion of the autophagosome with the lysosomes, and the degradation of materials in autolysosomes [39]. During autophagic flux, cytosolic LC3-I is conjugated to form LC3-II, and LC3-II is incorporated into the autophagosomal membrane. SQSTM1/p62 physically links autophagic cargo to the autophagic membrane [40, 41]. It is well established that lysosomal dysfunction leads to inhibition of autophagy in any type of cells, but cellular adaptation to abnormalities is cell type specific. For example, LC3-I accumulation and decreased LC3-II level have been described in GD macrophages [42], FD fibroblasts and lymphocytes, but conversely the basal LC3-II levels were increased in iPSC neuronal cells from GD L444P/L444P, N370S/N370S patients and in FD immortalized podocyte line [43–45] Our data are in agreement with reduced level of LC3-II in PBMCs from GD and FD patients similar to GD macrophages and FD lymphocytes (Table 1). Commonly, impairment of autophagy leads to accumulation of SQSTM1/p62. For example, GD fibroblasts and CBE treated neurons [46], as well as GBA knockout mice [38] demonstrated accumulation of SQSTM1/p62. In haematopoietic cell lineage, the SQSTM1/p62 was reported normal in GD macrophages [42]. Our data showed reduced level of SQSTM1/p62 in PBMCs from GD patients. SQSTM1/p62 accumulation has been reported in FD fibroblasts, but in the present study we observed reduced levels of SQSTM1/p62 in FD PBMCs (Table 1). However, in the cell and animal models, the observed occurrence may not adequately mimic what takes place in the human body. GD and FD patients exhibit chronic inflammation and elevated level of pro-inflammatory cytokines in blood [3, 47–49], and it is unclear how chronic inflammation impacts SQSTM1/p62 level. For example, patients with chronic obstructive pulmonary disease (COPD) with increased levels of Il-6, Il-8, TNF-α demonstrated decreased levels of SQSTM1/p62 in PBMCs [50]. Our RT-PCR study demonstrated reduced mRNA level of SQSTM1/p62 in GD1 and FD PBMCs.

**Table 1:** Autophagy, lysosomal and mitochondrial pathways in GD and FD with selected bibliography.

		PBMCs derived from patients		Other cell models	
		GD	FD	GD	FD
**Baseline**					
Autophagic vacuoles level		decreased	normal	Defect of autophagosome maturation was demonstrated in saposin C deficient and GBA mutant mouse models, drosophila models, iPSC-derived neuronal cells [51–53]. Decreased in gba-/- rat euroblastoma, cortical neurons [54], in vitro silencing mesenchymal stem cells [55].	Increased LC3 immunostaining in FD kidney tissue [43].
**Autophagy induction**					
SQSTM/p62	protein	decreased	decreased	Increased in iPS-derived neuronal cells [52], *gba*^-/-^ mouse neurons [38]. No change macrophages [42].	Increased in patient kidney tissue [43], histology staining.
	mRNA	decreased	decreased		
Autophagic vacuoles level	+RAP	no change	increased		No change in cultured FD podocytes cells [44].
**Autophagosome formation**					
Beclin 1	protein	elevated	elevated	Decreased in gba-/- rat neuroblastoma and cortical neurons [54]. Increased in GD fibroblasts [56].	
LC3A/B-II	protein	decreased	decreased	No change in iPS-derived neuronal cells [52], *in vitro* silencing bone marrow mesenchymal stem cells [55]. Decreased in *gba*^-/-^ rat neuroblastoma and cortical neurons [54], *gba*^-/-^ mouse neurons [38], macrophages [42], *in vitro* silencing amniotic fluid mesenchymal stem cells [55]. References with opposite results in fibroblasts [56, 57].	Decreased LC3A/B-I and no change level of LC3A/B-II in urine-derive FD cells [58], Increased in human GLA^-/-^ knockdown podocytes with absent response to rapamycin [44]. Increased total LC3 in patient kidney tissue [43] and *gla*^-/-^ mouse brain [59], histology staining.
	mRNA	normal	normal		
**Lysosomal markers**					
Lysosome levels		increased	normal	Increased in brains of *gba*^-/-^ mouse, drosophila model [53], fibroblasts [56], GD iPSC neurons [45].	Normal or increased in fibroblast cell lines [60].
LAMP1	protein	elevated	normal	References with opposite results in iPS-derived neuronal cells [45, 52].	Increased in *gla*^-/-^ mouse brain [59].
	mRNA	elevated	normal		
**Mitochondria**					
Mitochondrial function				Increased mitochondrial fragmentation, defective mitophagy in *gba*^-/-^ mouse neurons (GD2 model) [38].	Reduction of mitochondrial activity in FD fibroblasts [61].
Tfam	protein	increased	increased		
	mRNA	increased	increased		
ATP		normal	normal	mitochondria ATP consumer only in *gba*^-/-^ mouse neurons [38]. Reduced in GD fibroblasts [56].	Reduced level in FD fibroblast and heart [62].
mtDNA		increased	decreased		

**Fig 6:**
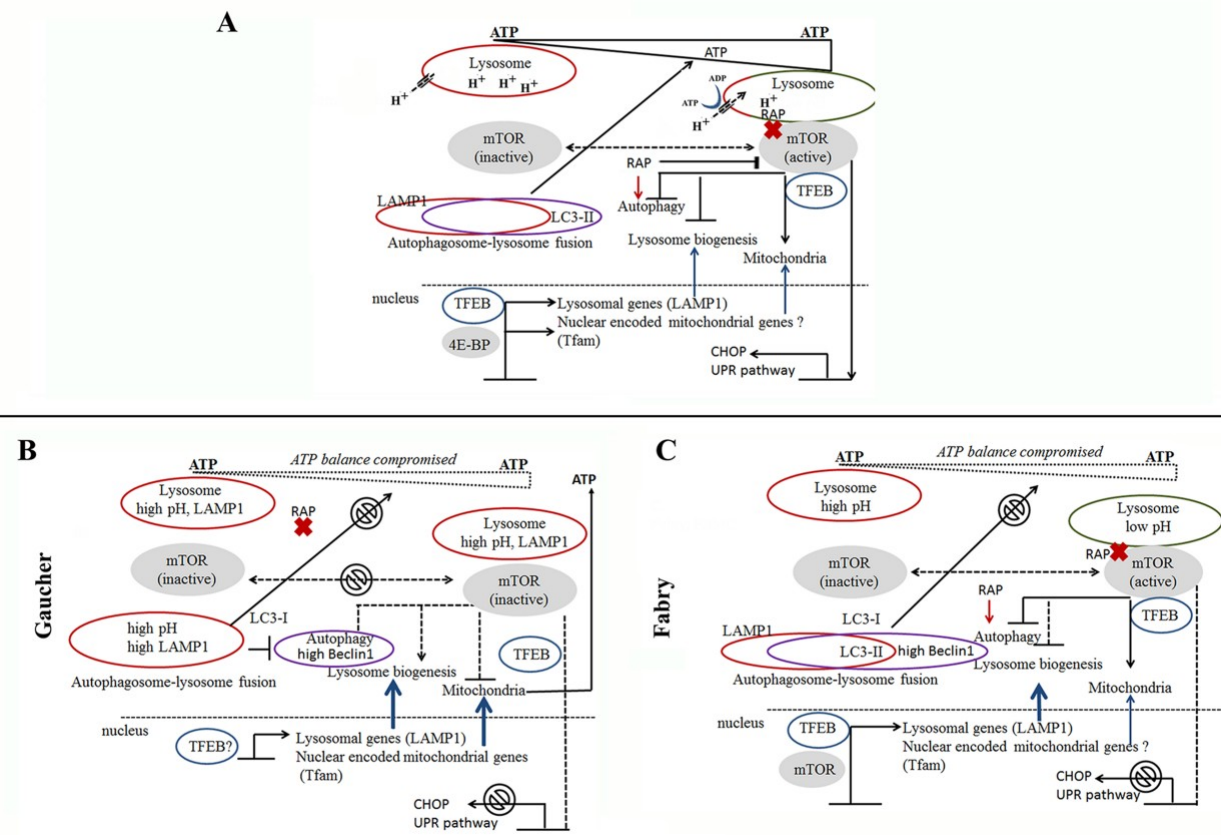
A working model of ALP dysfunction in GD and FD disrupt mTOR signalling pathway. **(A)** mTOR signalling pathway in normal cells. Nutrient availability and intracellular concentration of ATP are important for activation/inactivation of mTOR. Active form of mTOR is localized on lysosome, inhibits TFEB, autophagosome-lysosome fusion, regulates mitochondrial metabolism and activates UPR pathway. During nutrient-depletion, pH in lysosomes decreased and mTOR translocates to cytoplasm in inactive phase. TFEB migrates to the nucleus and activates expression of lysosomal genes. Transcription factor 4E-BP activates mitochondrial genes. Rapamycin inhibits mTOR activity (without releasing it from the lysosomal surface) and activates autophagosome-lysosome fusion. **(B).** mTOR signaling pathway in GD cells. Chronic elevation of lysosomal pH in GD prevents mTOR translocation to lysosomal surface and activation of the mTOR pathway. Moreover, lysosomal acidification obstructs autophagosome-lysosome fusion. Inhibition of mitochondrial metabolism leads to an imbalance in energy metabolism. Also, we postulate that TFEB migrates to the nucleus and activates the transcription of lysosomal genes and nuclear encoded mitochondrial genes. **(C)** mTOR signaling pathway in FD. In FD, α-Gal A deficiency leads to accumulation of Gb3 in lysosomes, partial inhibition of mTOR activity and deregulation of autophagosome-lysosome function and intracellular ATP balance.

It has been postulated that lysosomal dysfunction sets off a cascade of events that lead to autophagy abnormality [35, 61, 63]. The key regulator of autophagy-lysosomal and mitochondrial functions is the mTOR-dependent and mTOR-independent signaling pathways. Our data show that GCase and α–Gal A deficiency in lysosomes impair autophagic function and disrupt activation by rapamycin in PBMCs. These results link cellular pathology in GD and FD to the mTOR-dependent pathway. Multiple studies demonstrate that increased mTOR activity inhibits autophagy and generates lysosomal tubules that extrude from autolysosomes and form mature, functional lysosomes [64–66]. mTOR cycles between inactivation and activation phases to control the autophagosome-lysosome fusion process. Normally, the accumulation of amino acids in the lysosomal lumen triggers autophagy activation. Prior to this accumulation, mTOR is docked on the lysosomal surface in its active state inhibiting autophagy and lysosomal biogenesis (Fig 6, normal cells) [64, 67]. If mTOR reactivation does not occur autophagy is unable to proceed (Fig 6) [64]. There is limited information regarding the signaling pathway involved in mTOR cycling in LSDs *in vivo*; but *in vitro* studies suggest that mTOR activity is reduced in FD fibroblasts [35, 44], in *Drosophila melanogaster* [53] and others models of neuronopathic GD [46, 68, 69]. It is apparent that accumulation of metabolic substrates in the lysosomes inhibits autophagy-lysosome formation, and may lead to an inhibition of mTOR activation/inactivation cycle (Fig 6). Rapamycin, a pharmacological inhibitor of mTOR activity, stimulates autophagy initiation. Rapamycin does not affect mTOR localization to the lysosome; kinase-dead mTOR still localizes to lysosomes and activate autophagy (Fig 6) [70, 71]. Lack of mTOR inhibition by rapamycin may suggest that lysosomal dysfunction in GD prevents mTOR translocation to the lysosomal surface, and therefore, autophagy cannot proceed normally (Fig 6). Contrary to GD, where major pathology occurs in the peripheral blood and haematopoietic system, FD is leading to the accumulation of Gb3 mainly in the endothelium, resulting in pathology of the central nervous system, heart, kidney, and skin [72, 73]. Accumulation of Gb3 in Fabry podocyte cell culture leads to impaired autophagy and loss of mTOR kinase activity [44]. The markers of ALP are less drastically changed in PBMCs derived from FD patients compare with GD samples. This can explain milder haematopoitec system pathology in FD compared to GD. The functional integrity of lysosome and mitochondria is important for cell “health” [33]. Lysosomes play a pivotal role in autophagy-dependent lysosomal degradation of damaged mitochondria. Previous work with FD fibroblasts indicates that lysosomal dysfunction lead to mitochondrial dysfunction with a reduction of respiratory chain enzyme activity [61, 62]. Moreover, accumulation of Gb3 in lysosomes may lead to a deficit of sphingolipid content of the recycling lipids, which has a direct effect on the membrane lipid composition, for example, inner mitochondrial membrane [74]. Accumulation of GC in lysosomes also affects the mitochondrial function in GD. Mitochondrial dysfunction is reported in GD brain cells, in L444P/L444P fibroblasts and GD knockout mice [63, 75]. Small and fragmented mitochondria with reduced membrane potential and impaired aspiration was described in neurons from GD mouse model [38] and in fibroblasts derived from GD patients [56]. These observations imply that the dysfunctional lysosome leads to the accumulation of damaged mitochondria in the cells [8, 33, 76]. Our results demonstrated increased mt/nuclear DNA ratio in PBMCs derived from GD patients. This data supports the hypothesis that lysosomal dysfunction lead to accumulation of damaged mitochondria in GD cells.

While mitophagy inhibition could be an explanation for the mitochondrial dysfunction, this hypothesis may not be fully sufficient. The nutrition balance of cells aligned with ATP: ADP ratio controlling the mTOR activity. In response to energy depletion (low ATP levels), AMP-activated protein kinase (AMPK) and inhibits mTOR activity, induced autopahgy [77]. Our *in vivo* data suggest that GD and FD display mitochondrial dysfunction, including the lack of response to rapamycin. Therefore the impaired mTOR signaling pathway is likely to contribute to the mitochondrial dysfunction.

Defects of the autophagosome-lysosomal system do not affect the basal ATP levels in PBMCs derived from GD and FD patients, indicating a potential compensatory response that “turns on” another mechanism of mitochondrial regulation (Fig 6). mTOR regulates mitochondrial metabolism and biogenesis by promoting translation of nuclear-encoded genes, including Tfam [78]. Regulation of Tfam expression by two transcription factors TFEB or NRF1 can constitute a compensatory mechanism for stabilizing mitochondria. We observed a high variability of Tfam expression in PBMCs of GD and FD patients, but it showed an increasing trend. Sufficient transport of Tfam into mitochondria is corroborated by increased mtDNA copy numbers in GD, but not FD. Upon transport into the mitochondria, Tfam becomes active in the replication of mtDNA and generation of mtDNA gene products, which in turn, may stabilize energy balance in cells with chronic lysosomal pathology. On the other hand, accumulation of undigested mitochondria may explain increased mtDNA copy numbers.

ERT is the standard of care in patients with GD or FD. Although ERT greatly slows down the disease progression, difficulties in delivery to the crucial tissues validate the need to study cellular mechanism of lysosomal dysfunction *in vivo*. After intravenous infusion of the human recombinant enzyme, the physiological targeting of recombinant enzymes to lysosomes is mediated by mannose-6-phosphate receptors (M6PR) through activation of receptor mediated endocytosis [79, 80]. Lysosomal enzymes bind to M6PR in the trans-Golgi network then are trafficked to early and late endosomes and finally to the lysosomes after endosomal-lysosomal fusion [35]. However, dense dysfunctional lysosomes in PBMCs can impair trafficking of recombinant enzymes through the receptor-endocytosis pathway, and may impact the efficiency of ERT *in vivo*. Experiments comparing PBMCs before and after infusion, showed sufficient uptake of enzyme and the tendency of normalization of ALP (LAMP1 and LC3) and mitochondrial Tfam. *In vitro* studies confirmed our observation that during the first hour of ERT, autophagic-lysosomal function is normalized in PBMCs. However how long the ERT restores lysosomal function and whether or can correct cellular abnormalities is remain to be unclear.

## Supporting information

S1 Table Summary of patient demographic and clinical information. (DOCX)

S2 Table Primers used for RT-PCR. (DOCX)

S1 Fig Evaluation of autophagy-lysosomal markers in patient-derived PBMCs. **(A)** PBMCs from healthy control and patients with GD (type 1 and 3) were stained with Cyto-ID autophagy detection kit. The graph shows the relative levels of autophagosome vesicle formation in GD samples with mutations N370S/R463C, N370S/L444P, or N370S/N370S from individual patients. **(B)** Same as (A) for FD patients with mutations C982G/G328R, R49P, or exon 2 deletion. **(C)** Representative western blots of PBMC showing Beclin1 and LAMP1 protein expression levels in GD (type 1 and 3), FD, and control samples. **(D)** Quantification of LC3-II protein level from control (n = 8), GD1 (n = 3), GD3 (n = 4) and FD (n = 4) samples after western blot. Values normalized to control group. *p<0.*05* Student’s T-Test.
(TIF)

S2 Fig The effect of rapamycin (RAP) on LAMP1 and Beclin 1 levels in GD and FD. **(A)**, Inhibition of mTOR by rapamycin was analysed by incubating PBMCs from healthy control and primary fibroblast after 48 h starvation were treated 3h with the 10 nM rapamycin. The whole-cells extracts were immunoblotted with phospho-mTOR (Ser2448) and mTOR antibodies as indicated. (B), PBMCs deriviedfrom GD type 1 and 3, and FD were treated 3h with 10 nM rapamycin (RAP). Western blot showing Beclin1 and LAMP1 protein expression in PBMCs derived from control, GD type 1 and type 3 patients with N370S/L444P and L444P/L444P mutations, and FD patients with G:A deletion c.718-718 del. Membranes were stained with Ponceau S for normalization. **(C)** PBMCs from healthy controls (n = 11), GD type 1 (n = 15), and FD (n = 13) were measured with a Ser2448 and total mTOR sandwich ELISA kits. Samples were measured in triplicates. Values normalized to the average control group. *p<0.*05* Student’s T-Test.
(TIF)

S3 Fig Mitochondrial function is affected in GD and FD. **(A)** ATP levels measured in PBMCs derived from healthy control, GD1, GD3 and FD patients using CellTiter-Glo luminescent cell viability assay. Each column represents an individual patient with known mutations. **(B)** Relative level of Tfam normalized to actin in healthy control and in GD1 patients with different *GBA* mutations. Values are the *average ± SEM*. **(C)** Tfam protein expression in PBMC derived from healthy control, GD1 and FD patients (left panel). The right panel showing representative western blot of Tfam in PBMCs derived from control subjects and GD type 3 patients.
(TIF)

S4 Fig GBA, LAMP1, LC3A/B and Tfam protein levels in PBMC. PBMCs were collected before (Pre) and after (Post) ERT infusion from GD1 patients. Representative western blots show GBA (A), LAMP1 (B), LC3-I/II (C) and Tfam (D) protein expression in PBMCs of healthy and GD1 patient before and after ERT infusion (top). Ponceau S from the same membranes (bottom).
(TIF)

S5 Fig Uptake and effect of ERT in THP1 cells. **(A)** Intracellular enzymatic activity of THP1 cells treated with the recombinant enzyme at concentrations of 500, 50, 5, 0.5, or 0.05 μg/ml. **(B)** Percentage of autophagosomes in THP1 cells treated with Cerezyme and velaglucerase alpha at concentrations of 500, 50, 5, 0.5, or 0.05 μg/ml relative to an untreated control. **(C)** Autophagosome staining of THP1 cells treated with rhGCase at concentrations of 500, 50 and 5 μg/ml. Staining was performed using Cyto-ID autophagy kit (green color).
(TIF)
