# Supplementary material for: Impaired autophagic and mitochondrial functions are partially restored by ERT in Gaucher and Fabry diseases
Source: PLoS One. 2019 Jan 11;14(1):e0210617. doi: 10.1371/journal.pone.0210617 (PMC6329517; doi:10.1371/journal.pone.0210617)
Supplement: S1 Table — (DOCX) [file pone.0210617.s001.docx]

S1 Table. Summary of patient demographic and clinical information.

|  | **Age** | **Gender** | **Genotype** | **Ethnicity** |
| --- | --- | --- | --- | --- |
| GD type 1 | | | | |
| 1 | 37 | F | N370S/R463C | Caucasian |
| 2 | 35 | F | N370S/R463C | Caucasian |
| 3 | 58 | F | N370S/L444P | Caucasian |
| 4 | 33 | M | N370S/unknown | Hispanic |
| 6 | 24 | M | N370S/L444P | Hispanic |
| 6 | 16 | M | N370S/L444P | Hispanic |
| 7 | 27 | F | N370S/L444P | Hispanic |
| 8 | 63 | F | N370S/R463C | Caucasian |
| 9 | 56 | F | N370S/N370S | Caucasian |
| 10 | 46 | F | N370S/N370S | Ashkenazi |
| 11 | 72 | F | unknown | Caucasian |
| 12 | 64 | F | N370S | Declined |
| 13 | 51 | F | L444P/unknown | Caucasian |
| 14 | 56 | F | N370S/N370S | Ashkenazi |
| 15 | 59 | M | N370S/N370S | Caucasian |
| 16 | 60 | F | N370S/unknown | Ashkenazi |
| 17 | 50 | M | N370S/L444P | Ashkenazi |
| 18 | 41 | F | N370S/L444P | N. European |
| 19 | 7 | F | N370S/L444P | Caucasian |
| 20 | 77 | M | N370S/L444P | Caucasian |
| 21 | 62 | F | N370S/R463C | Caucasian |
| 22 | 35 | F | N370S/R120Q | Ashkenazi |
| 23 | 64 | F | N370S/unknown | Declined |
| 24 | 47 | F | N370S/unknown | Caucasian |
| 25 | 49 | F | R257Q | Caucasian |
| 26 | 51 | F | I448C/L444P | Caucasian |
| 27 | 58 | F | 1226G | Caucasian |
| 28 | 72 | F | P450 2D6 | Caucasian |
| 29 | 33 | M | unknown | Hispanic |
| 30 | 48 | F | unknown | Denied |
| 31 | 40 | M | unknown | Ashkenazi |
| 32 | 68 | M | unknown | Denied |
| 33 | 70 | M | N370S/N370S | Ashkenazi |
| GD type 3 | | | | |
| 1 | 16 | F | L444P/L444P | Hispanic |
| 2 | 19 | M | L444P/L444P | Hispanic |
| 3 | 38 | F | L444P/L444P | Caucasian |
| 4 | 12 | F | L444P/L444P | Hispanic |
| 5 | 16 | M | N188S/S107L | Other |
| FD | | | | |
| 1 | 56 | M | Ex. 2 deletion | Caucasian |
| 2 | 52 | M | unknown | Declined |
| 3 | 9 | M | c.718_719delAA | Caucasian |
| 4 | 20 | M | c.879C>T | Caucasian |
| 5 | 20 | M | unknown | Hispanic |
| 6 | 22 | M | unknown | Hispanic |
| 7 | 38 | M | c.1033_1034delTC | Caucasian |
| 8 | 61 | M | 1188del1 | Caucasian |
| 9 | 58 | M | C2233Y | Caucasian |
| 10 | 18 | M | unknown | Hispanic |
| 11 | 52 | M | unknown | Hispanic |
| 12 | 26 | M | c.806T>A | African American |
| 13 | 12 | M | 2 mutation AIRE gene | Caucasian |
| 14 | 13 | M | R49P | Caucasian |
| 15 | 27 | M | c.256delT | Caucasian |
| 16 | 31 | M | No confirmation | Caucasian |
| 17 | 25 | M | No confirmation | Caucasian |
| 18 | 34 | M | c.1032-1033delTC | Caucasian |
| 19 | 43 | M | c.1032-1033delTC | Caucasian |
| 20 | 39 | M | c.1032-1033delTC | Denied |
| 21 | 17 | M | No confirmation | Caucasian |
| 22 | 23 | M | No confirmation | Caucasian |
| 23 | 17 | F | C.718_719DELAA | Caucasian |
| 24 | 39 | F | C.718_719DELAA | Caucasian |
| 25 | 30 | F | R49P | Caucasian |
| 26 | 44 | F | C982G/G328R | Caucasian |
| 27 | 17 | F | R49P | Caucasian |
