# Supplementary material for: Impaired autophagic and mitochondrial functions are partially restored by ERT in Gaucher and Fabry diseases
Source: PLoS One. 2019 Jan 11;14(1):e0210617. doi: 10.1371/journal.pone.0210617 (PMC6329517; doi:10.1371/journal.pone.0210617)
Supplement: S2 Table — (DOCX) [file pone.0210617.s002.docx]

**Supplemental Table 2. Primers used for RT-PCR.**

| **Name** | **Abbreviation** | ***Sequence (5’-3’)*** |
| --- | --- | --- |
| Lysosomal associated membrane protein 1 | LAMP1 | *5’-ctgcctttaaagctgccaac-3’* |
|  |  | *5’-tgttctcgtccagcagacac-3’* |
| Microtubular associated protein 1 light chain 3 alpha | LC3A/B  (MAPPL1L3A) | *5’-gatgtccgacttattcgagagc-3’* |
|  |  | *5’-ttgagctgtaagcgccttcta-3’* |
| Heat shock protein family A (Hsp70) member 5 | HSPA5  (GRP-78) | *5’-catcaagttcttgccgttca-3’* |
|  |  | *5’-atgtctttgtttgcccacct-3’* |
| Glyceradehyde-3-phosphate dehydrogenase | GAPDH | *5’-tcatctctgccccctctgct-3’* |
|  |  | *5’-cgacgcctgcttcaccacct-3’* |
| Hypoxanthine phosphoribosyltransferase 1 | HPRT | *5’-tatggcgacccgcagccct-3’* |
|  |  | *5’-catctcgagcaagacgttcag-3’* |
