# Supplementary figures and images for: Impaired autophagic and mitochondrial functions are partially restored by ERT in Gaucher and Fabry diseases
Source: PLoS One. 2019 Jan 11;14(1):e0210617. doi: 10.1371/journal.pone.0210617 (PMC6329517; doi:10.1371/journal.pone.0210617)

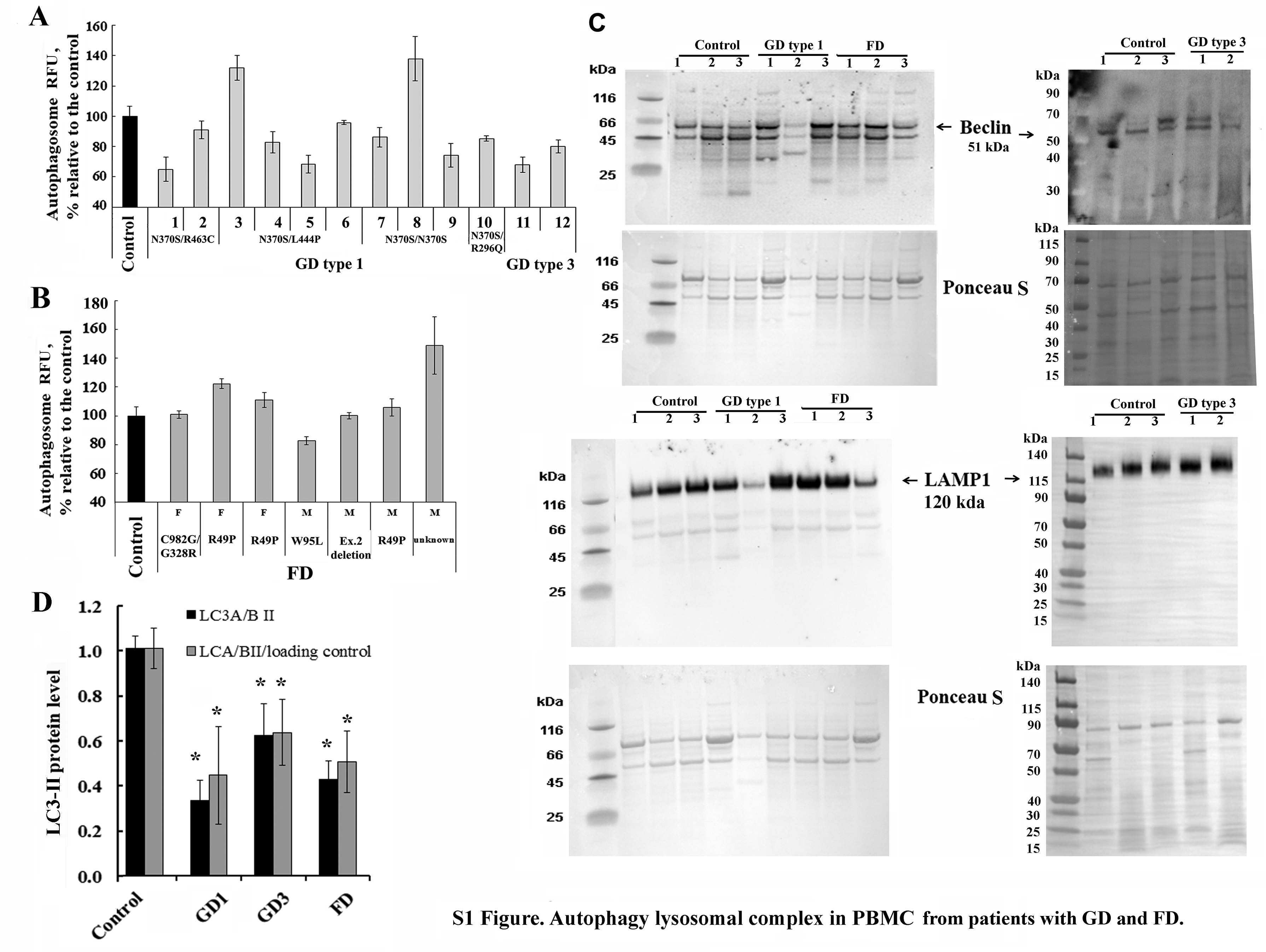

Supplement: S1 Fig — (A) PBMCs from healthy control and patients with GD (type 1 and 3) were stained with Cyto-ID autophagy detection kit. The graph shows the relative levels of autophagosome vesicle formation in GD samples with mutations N370S/R463C, N370S/L444P, or N370S/N370S from individual patients. (B) Same as (A) for FD patients with mutations C982G/G328R, R49P, or exon 2 deletion. (C) Representative western blots of PBMC showing Beclin1 and LAMP1 protein expression levels in GD (type 1 and 3), FD, and control samples. (D) Quantification of LC3-II protein level from control (n = 8), GD1 (n = 3), GD3 (n = 4) and FD (n = 4) samples after western blot. Values normalized to control group. *p<0.05 Student’s T-Test. (TIF) [file pone.0210617.s003.tif]

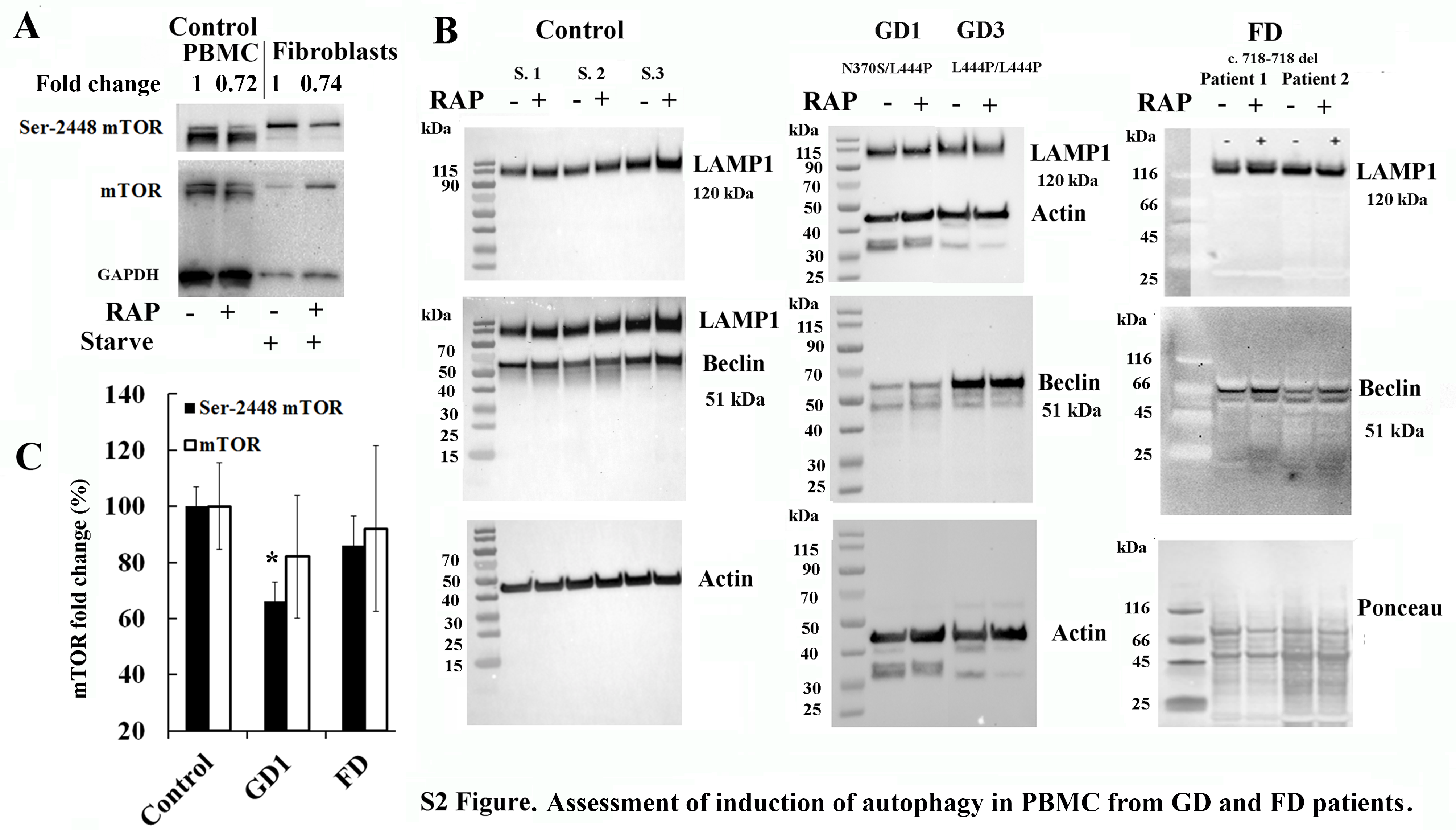

Supplement: S2 Fig — (A), Inhibition of mTOR by rapamycin was analysed by incubating PBMCs from healthy control and primary fibroblast after 48 h starvation were treated 3h with the 10 nM rapamycin. The whole-cells extracts were immunoblotted with phospho-mTOR (Ser2448) and mTOR antibodies as indicated. (B), PBMCs deriviedfrom GD type 1 and 3, and FD were treated 3h with 10 nM rapamycin (RAP). Western blot showing Beclin1 and LAMP1 protein expression in PBMCs derived from control, GD type 1 and type 3 patients with N370S/L444P and L444P/L444P mutations, and FD patients with G:A deletion c.718-718 del. Membranes were stained with Ponceau S for normalization. (C) PBMCs from healthy controls (n = 11), GD type 1 (n = 15), and FD (n = 13) were measured with a Ser2448 and total mTOR sandwich ELISA kits. Samples were measured in triplicates. Values normalized to the average control group. *p<0.05 Student’s T-Test. (TIF) [file pone.0210617.s004.tif]

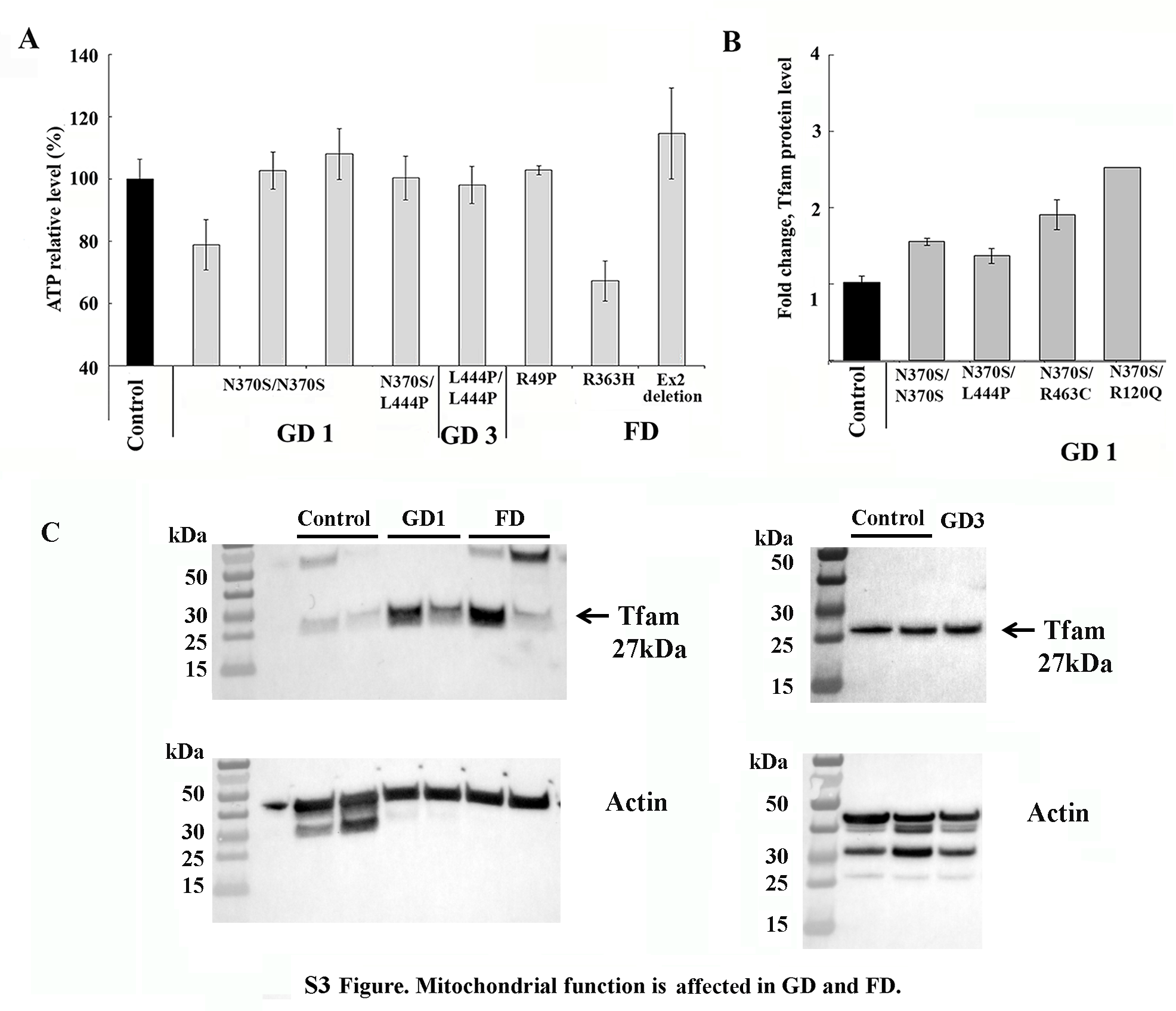

Supplement: S3 Fig — (A) ATP levels measured in PBMCs derived from healthy control, GD1, GD3 and FD patients using CellTiter-Glo luminescent cell viability assay. Each column represents an individual patient with known mutations. (B) Relative level of Tfam normalized to actin in healthy control and in GD1 patients with different GBA mutations. Values are the average ± SEM. (C) Tfam protein expression in PBMC derived from healthy control, GD1 and FD patients (left panel). The right panel showing representative western blot of Tfam in PBMCs derived from control subjects and GD type 3 patients. (TIF) [file pone.0210617.s005.tif]

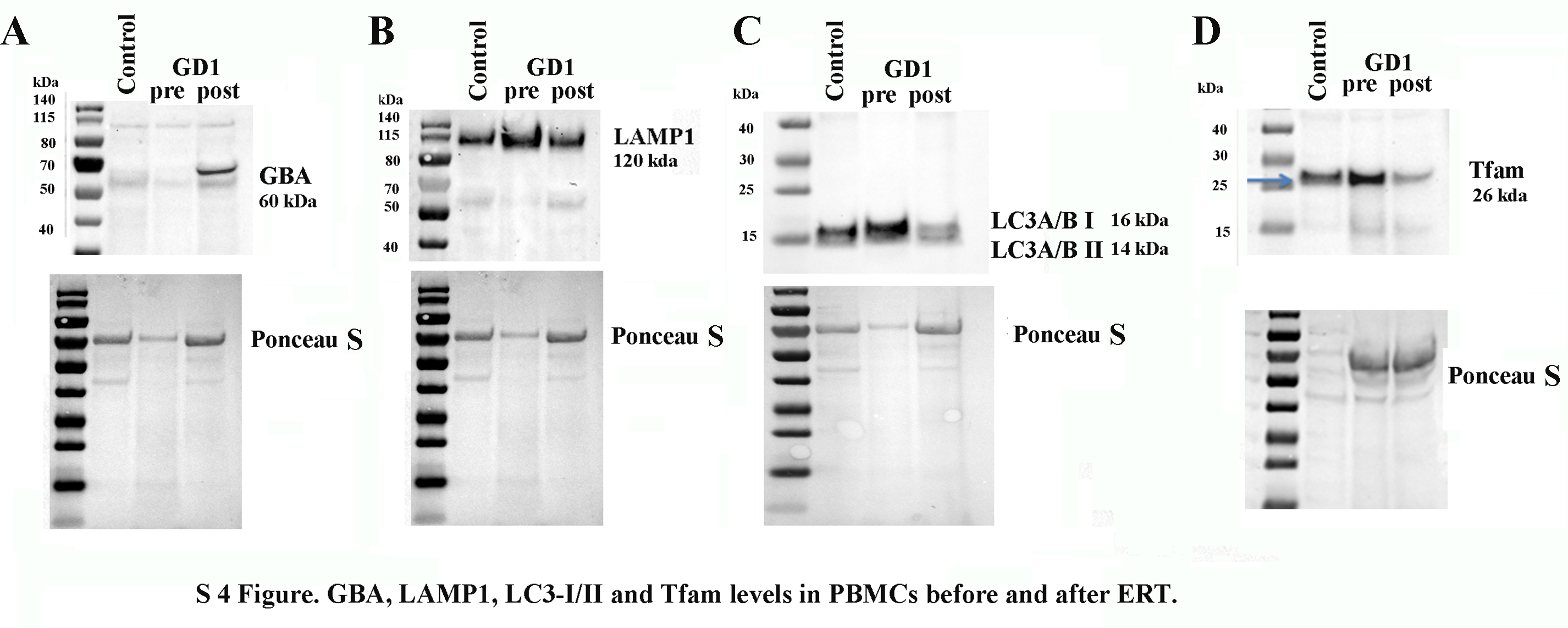

Supplement: S4 Fig — PBMCs were collected before (Pre) and after (Post) ERT infusion from GD1 patients. Representative western blots show GBA (A), LAMP1 (B), LC3-I/II (C) and Tfam (D) protein expression in PBMCs of healthy and GD1 patient before and after ERT infusion (top). Ponceau S from the same membranes (bottom). (TIF) [file pone.0210617.s006.tif]

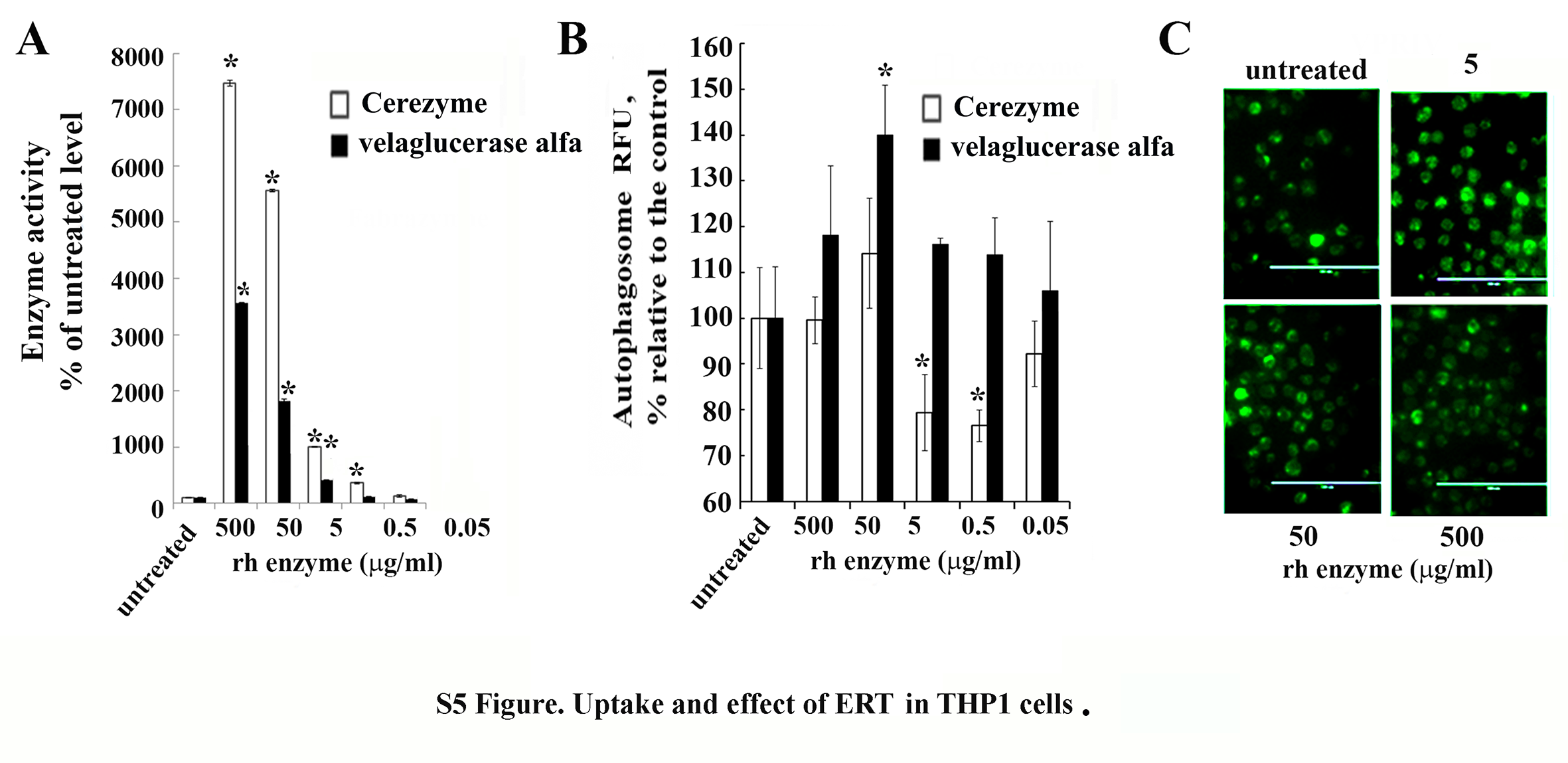

Supplement: S5 Fig — (A) Intracellular enzymatic activity of THP1 cells treated with the recombinant enzyme at concentrations of 500, 50, 5, 0.5, or 0.05 μg/ml. (B) Percentage of autophagosomes in THP1 cells treated with Cerezyme and velaglucerase alpha at concentrations of 500, 50, 5, 0.5, or 0.05 μg/ml relative to an untreated control. (C) Autophagosome staining of THP1 cells treated with rhGCase at concentrations of 500, 50 and 5 μg/ml. Staining was performed using Cyto-ID autophagy kit (green color). (TIF) [file pone.0210617.s007.tif]
